# Darwin’s legacy to rove beetles (Coleoptera, Staphylinidae): A new genus and a new species, including materials collected on the Beagle’s voyage

**DOI:** 10.3897/zookeys.379.6624

**Published:** 2014-02-12

**Authors:** Stylianos Chatzimanolis

**Affiliations:** 1 Department of Biological and Environmental Sciences, University of Tennessee at Chattanooga, 615 McCallie Ave, Dept. 2653, Chattanooga, Tennessee 37403, USA

**Keywords:** Argentina, Neotropical, South America, Staphylininae, Staphylinini, Xanthopygina

## Abstract

A species of xanthopygine rove beetles is described and figured here as *Darwinilus sedarisi* **gen. n.** and **sp. n.** The holotype was collected by Charles Darwin in Bahía Blanca, Argentina on the Beagle’s voyage. The contributions of Charles Darwin to rove beetle systematics are summarized briefly.

## Introduction

Charles Darwin was an avid beetle collector and his contributions to the study of entomology have been extensive (Stephens 1827–1845; Waterhouse 1879; Champion 1918; Kritsky 1981; Smith 1987). Darwin’s collecting efforts on the Beagle’s voyage (1831–1836) were important because he brought back to the United Kingdom specimens from places that had not been sampled before. Darwin kept meticulous notes on the specimens he collected and those notes are known as “Insect Notes” (kept at the Entomology Library of the Natural History Museum, London) and “Insects in Spirits of Wine” (kept at the Cambridge University Library). Smith (1987) provided annotated versions of those notes giving details on the taxonomy of the specimens collected and whether or not these specimens still exist in collections.

Based on the annotated Insect Notes (Smith 1987) we know that Darwin had at least 14 collecting events that included rove beetles (Table 1). These include species in the subfamilies Aleocharinae, Microsilphinae, Oxytelinae, Pselaphinae, Scaphidiinae, Scydmaeninae and Staphylininae. Until now, five new species of rove beetles had been described based on Darwin materials and most of those are still considered valid species. Four more species of rove beetles are currently known from Darwin’s collecting efforts but the type materials of these species did not include Darwin’s specimens. Additionally, Smith (1987) did not find specimens for some of the collecting events that included rove beetles.

**Table 1.** Checklist of rove beetles collected by Charles Darwin on the Beagle’s Voyage. The list has been extracted from Smith (1987) with updates on the taxonomy. Date refers to the date of the collecting event as documented by Darwin. Months are given in roman numerals (when available). Specimen no. refers to the collecting event number given by Darwin.

Date	No.	Locality	Subfamily: Tribe: Subtribe	Species	Comments	Reference
1832-ii-16	229	St. Paul’s Rocks, Brazil			Specimen not found; hypothesized by Smith (1987) to be either *Philonthus* or *Quedius*.	Smith (1987)
1832-iv	415	Rio de Janeiro, Brazil	Scaphidiinae: Scaphisomatini	*Scaphisoma elongatum* Waterhouse, 1879	Species described from Darwin specimen.	Waterhouse (1879)
1832-ix	708	Bahía Blanca, Argentina	Staphylininae: Staphylinini: Xanthopygina	*Darwinilus sedarisi* Chatzimanolis, gen. n. and sp. n.	Specimen listed as “not found” in Smith (1987); genus and species described from Darwin specimen.	this paper
1832-xii-20	906	Navarin Is., Chile	Aleocharinae: Oxypodini	*Nordenskjoldella flavitarsis* Enderlein, 1912	Type material not based on Darwin specimen.	Enderlein (1912); Champion (1918)
1833-iii	1151	Tierra del Fuego	Aleocharinae: Homalotini	*Leptusa (Halmaeusa) atriceps* (Waterhouse, 1875)	Originally described as *Phytosus darwini* Waterhouse 1879 and was based on Darwin specimen.	Waterhouse (1879); Steel (1964)
1834-i	2002	Port St Julian [Puerto San Julián], Argentina			Specimens not found.	Smith (1987)
1834	2369	Archipelago of Chiloé, Chile	Microsilphinae	*Microsilpha ocelligera* (Champion, 1918)	Originally described in *Micragyrtes*; type materials based on Darwin materials.	Champion (1918); Newton and Thayer (1995)
1834	2371	Archipelago of Chiloé, Chiloé Is., Chile	Aleocharinae: Oxypodini	*Polylobus darwini* Bernhauer, 1935	Species described from Darwin specimen.	Bernhauer (1935)
1834-xii	2424	Archipelago of Chronos, Chile			Specimens not found; Darwin commented that “Pselaphidae and small Staphylinidae the most abundant insects”	Smith (1987)
1835	3426	Galapagos Archipelago, San Cristóbal Is., Ecuador	Staphylininae: Staphylinini	*Creophilus galapagensis* Clarke, 2011	Type material not based on Darwin specimen.	Clarke (2011)
1835-ii	3445	Hobart Town, Tasmania, Australia			Specimens not found; hypothesized by P. Hammond to be *Creophilus erythrocephalus* F.	Smith (1987)
1835-ii	3524	Hobart Town, Tasmania, Australia	Scaphidiinae: Scaphisomatini	*Scaphisoma instabile* Lea, 1926	Lectotype not based on Darwin specimen.	Lea (1926); Löbl (1977)
1836-vii	3730	St. Helena	Scydmaeninae: Cyrtoscydmini	“*Anthicus wollastoni*” Waterhouse, 1879	The type was based on Darwin material; not Anthicidae but Scydmaeninae (in *Euconnus*) according to Smith (1987).	Waterhouse (1879); Champion (1895)
1836-vii	3730	St. Helena	Oxytelinae: Oxytelini	*Oxytelus alutaceifrons* Wollaston, 1877	Type material not based on Darwin specimen.	Wollaston (1877); Waterhouse (1879)

Over the last several years, I have been working towards revising all genera in the rove beetle subtribe Xanthopygina, a group of large and colorful rove beetles distributed in the New World tropics (Chatzimanolis 2014). While examining specimens for the review of *Trigonopselaphus* Gemminger and Harold (Chatzimanolis in preparation), I noticed a specimen borrowed from the Natural History Museum (London) that had serrate antennae, an atypical morphological feature in rove beetles. Upon further inspection, I realized that the specimen belonged to an undescribed genus and that it was Charles Darwin who had collected it on the Beagle’s voyage. In this paper I describe this and one additional conspecific specimen as a new genus and species of Xanthopygina, the second new genus of rove beetles to be described from Beagle’s expedition materials.

## Materials and methods

Specimens were studied using an Olympus SZX10 dissecting microscope. Specimens examined were loaned from the Natural History Museum, London (BMNH; Roger Booth) and the Museum für Naturkunde der Humboldt Universität (ZMHB; M. Uhlig, B. Jaeger). The 181-year old Darwin specimen was relaxed carefully using the steam method described in a Natural History Museum (London) blog post by curator Beulah Garner, (http://www.nhm.ac.uk/natureplus/blogs/beetles/2011/11/05/steamy-beetles-or-whats-the-point). The paratype was already dissected when I received the specimen from ZMHB. Some aspects of the morphology (e.g., extensive details on mouthparts) were not described due to the fragile state (and at the same time high scientific value) of both specimens. Photographs were taken using a Visionary Digital Passport system with a Canon EOS 40D. Final images were automontaged using Helicon Focus 4.2.9 Pro (http://www.heliconsoft.com/heliconfocus.html). Total length of the specimens is measured from the anterior margin of frons to the posterior margin of segment VIII; width: length measurements were made on the widest: longest part of the structure. Measurements were made with an ocular micrometer. The comparison of the length of the parameres and the median lobe excludes the bulbous basal portion of the median lobe. For type label data, the slash “/” separates different labels. Morphological terminology follows Ashe and Chatzimanolis (2003) and other recent revision of Xanthopygina (Chatzimanolis 2004, 2008, 2012; Chatzimanolis and Ashe 2009). In this paper I follow the phylogenetic species concept as outlined by Wheeler and Platnick (2000).

## Taxonomy

### Family Staphylinidae Latreille, 1802
Subfamily Staphylininae Latreille, 1802
Tribe Staphylinini Latreille, 1802
Subtribe Xanthopygina Sharp, 1884

#### Darwinilus

Chatzimanolis gen. n.

http://zoobank.org/BD229C1A-4D45-4BF5-B780-52CA5C2720B2

http://species-id.net/wiki/Darwinilus

##### Type species.

*Darwinilus sedarisi* Chatzimanolis, sp. n.

##### Diagnosis.

*Darwinilus* can be distinguished from all other Xanthopygina genera by the combination of the following characters: a) serrate antennae (antennomeres 5–11; antennomeres 6–10 asymmetrical in *Terataki* Chatzimanolis, *Triacrus* Nordmann and *Trigonopselaphus* but not as in *Darwinilus*); b) clypeus with shallow emargination; c) protibia strongly curved and d) absence of porose structure on abdominal sternite VII in males. *Darwinilus* is probably closely related to the genera *Terataki* Chatzimanolis and/or *Haematodes* Laporte and *Weiserianum* Bernhauer but can be easily distinguished from these genera by the presence of serrate antennae in *Darwinilus* and the lack of porose structure on abdominal sternite VII in males (present in *Terataki*, *Haematodes* and *Weiserianum*).

##### Description.

**Figure 1.**
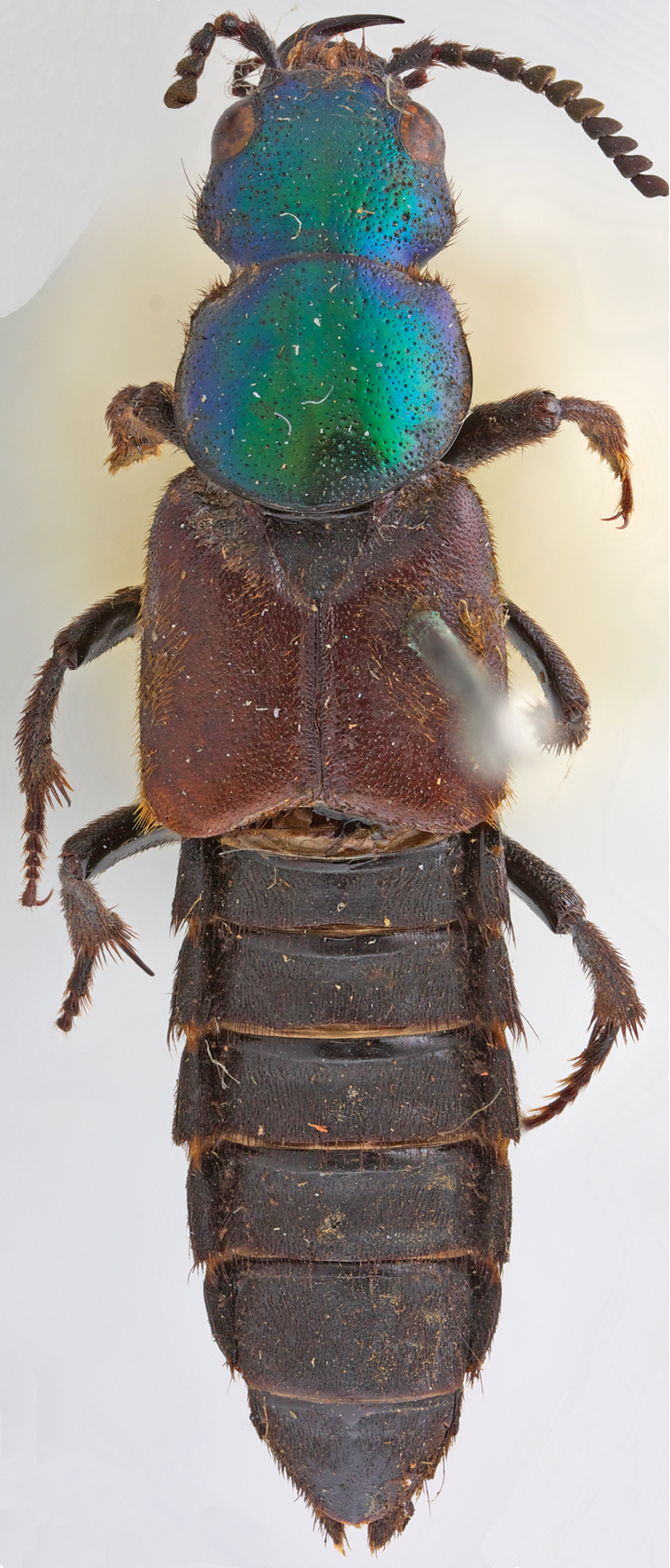
Habitus of the holotype of *Darwinilus sedarisi* Chatzimanolis, sp. n. Total length = 21.5 mm Image Copyright Natural History Museum (London).

**Figures 2–5. F2:**
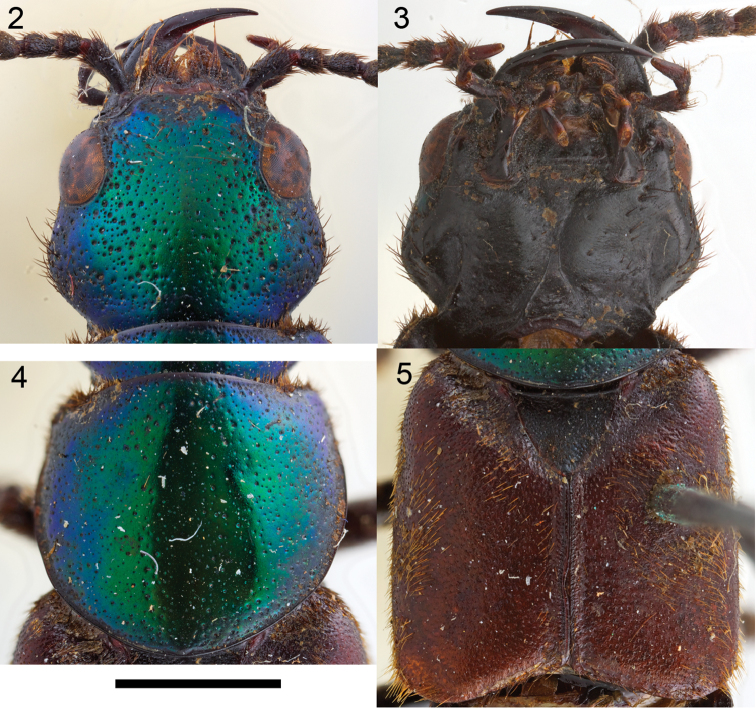
Head and thorax of the holotype of *Darwinilus sedarisi* Chatzimanolis, sp. n. **2** Head, dorsal view **3** Head, ventral view **4** Pronotum **5** Elytra. Scale = 2.2 mm Image Copyright Natural History Museum (London).

**Figures 6–7. F3:**
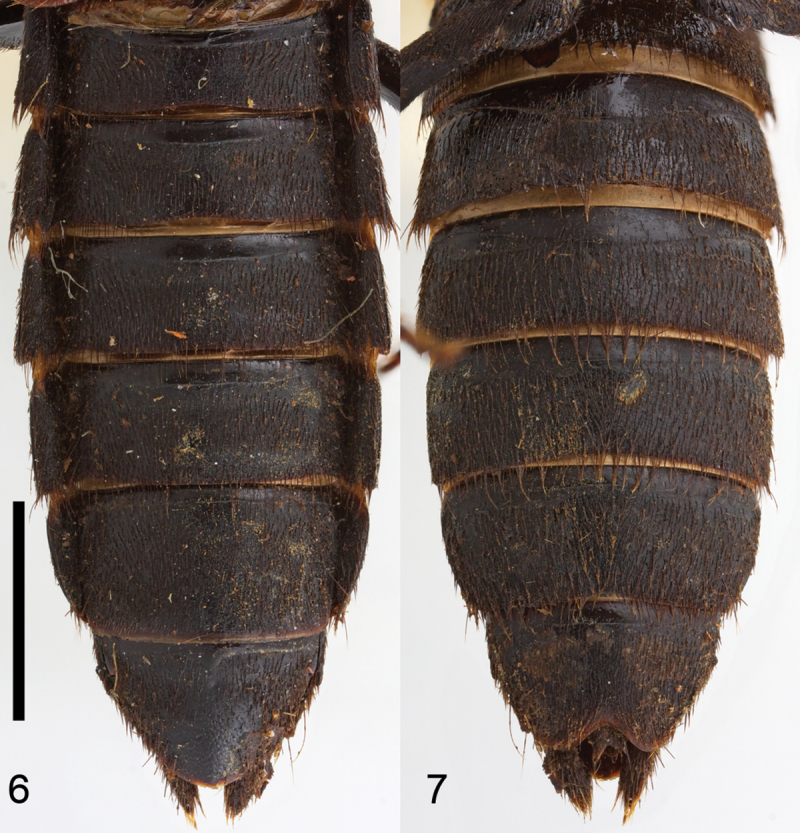
Abdomen of the holotype of *Darwinilus sedarisi* Chatzimanolis, sp. n. **6** Dorsal view **7** Ventral view. Scale = 3 mm Image Copyright Natural History Museum (London).

**Figures 8–9. F4:**
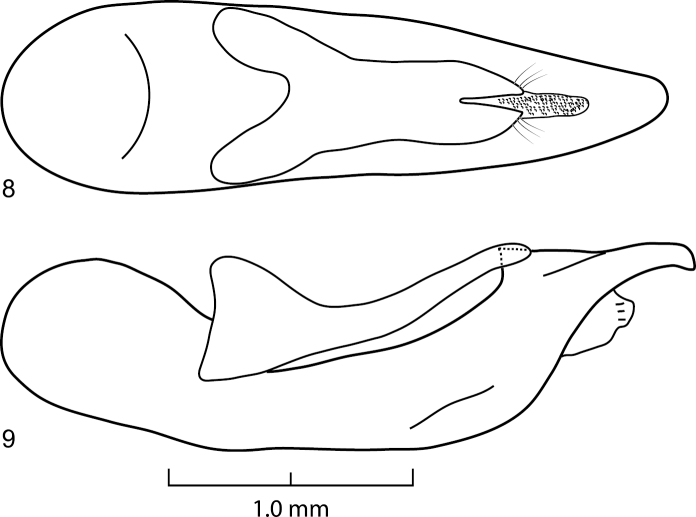
Aedeagus of *Darwinilus sedarisi* Chatzimanolis, sp. n. **8** Dorsal view **9** Lateral view.

Habitus as in Fig. 1, body large, robust. Head hexagonal in shape (Figs 2–3), widest at temples. Eyes medium-sized, positioned anteriorly, distance between eyes as wide as twice length of eye. Postoccipital suture and ventral basal ridge present; presence of infraorbital ridge not clear but ridge situated between postmandibular ridge and gular suture extends from posterior to middle part of gena; postmandibular ridge present and prominent; gular sutures converging medially; without neck (no nuchal ridge). Epicranium with large prominent macrosetae around lateral margins. Anteclypeus expanded, clypeus with small v-shaped emargination medially. Antennae serrate, 11–segmented; antennomeres 1–3 with several rows of macrosetae; antennomeres 4–11 covered with microtrichiae. Mouthparts with labrum medially emarginate to its base. Mandibles curved, elongate, symmetrical, with prominent fold extending from base to near middle; right mandible with at least one prominent tooth; prostheca setose. Maxilla with galea and lacinia setose; maxillary palpi 4-segmented; palpomeres with several large setae; P_1_ short; P_2_–P_4_ elongate; P_2_–P_3_ curved, wider distally; P_2_ 2.2 times as long as P_1_; P_3_ shorter than P_2_; P_4_ subequal to P_3_, rounded apically. Labium with mentum having two anterolateral setae on each side; ligula short, entire; labial palpi 3-segmented; P_1_ subequal to P_2_; P_2_ widest anteriorly, with many large setae; P_3_ elongate, longer than P_2_, securiform [but not as dilated as in *Zackfalinus* Chatzimanolis or *Dysanellus* Bernhauer; see Chatzimanolis 2012]. Pronotum slightly wider than head; with small translucent postcoxal process; pronotal hypomeron expanded; superior and inferior marginal lines of hypomeron separate throughout their length and superior line fully visible from above (typical of Xanthopygina). Anterolateral corners of pronotum prominent. Pronotum (Fig. 4) with microsculpture and punctures of various sizes; with prominent macrosetae along margins. Basisternum with transverse microsculpture and various setae; anterior marginal depression present; sternacostal ridge present; furcasternum without carina. Elytra (Fig. 5) longer than pronotum; with long yellow macrosetae, especially prominent at lateral and posterior margins. Elytra depressed near mesoscutellum. Hind wings fully developed. Mesoventrite without median carina or mesoventral process; metaventrite with transverse microsculpture and uniform medium-sized punctation; metaventral process small, triangular. Legs with tarsal segmentation 5-5-5; tibia with ctenidium and several rows of small spurs; meso- and metatibia with two long apical spurs, spurs as long as basitarsus; protibia strongly curved; meso- and metatibia slightly curved. Protarsus enlarged in males [no females are known]; meso- and metatarsi not enlarged; empodium with two setae. Abdomen (Figs 6–7) with abdominal tergites III–V with anterior basal carina but without curved (arch-like) ridge and without accessory basal lines. Abdominal sternite VII in males without porose structure. Male genitalia (Figs 8–9) typical of Xanthopygina; aedeagus with long median lobe; paramere partially divided distally.

##### Etymology.

The genus name is derived from the word “Darwin” in honor of Charles Darwin who collected the beetle during the voyage of the Beagle. The name is masculine.

#### Darwinilus sedarisi

Chatzimanolis sp. n.

http://zoobank.org/6AB0C47D-5A4B-4D59-AB99-E188FB1E95D2

http://species-id.net/wiki/Darwinilus_sedarisi

Figs 1 [Fig F2] [Fig F3] [Fig F4] –10

##### Type locality.

Bahía Blanca, Argentina.

##### Holotype.

Male, dry pinned, with labels as follows: “B. Blanca” / “708” / “Darwin Coll. 1885.-119.” / “Bahía Blanca, Argentina. C. Darwin.” / “?*Trigonopselaphus* A. Solodovnikov det. 2007” / “Holotype *Darwinilus sedarisi* Chatzimanolis des. Chatzimanolis 2013”. Darwin arrived on Bahía Blanca on September 6, 1832 and departed on October 17, 1832 according to Barlow (1967). The specimen was collected in September according to the Insect Notes that Darwin kept (Smith 1987). The holotype shows evidence of prior damage since several body parts have been reattached with non water-soluble glue. Deposited in BMNH. **Paratype** (1) male: **Argentina**, Córdoba, Río Cuarto, Breuer coll. (ZMHB).

**Figure 10.**
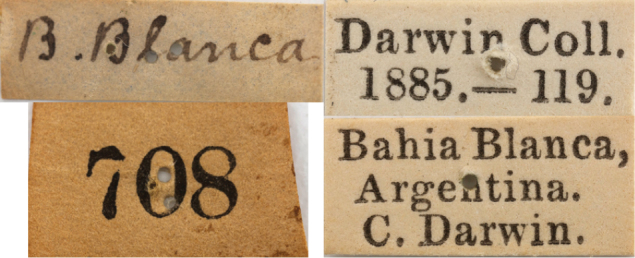
Original BMNH labels for the holotype of *Darwinilus sedarisi* Chatzimanolis, sp. n. Image Copyright Natural History Museum (London).

##### Diagnosis.

As for the genus.

##### Description.

Body length 20.0–21.5 mm. Coloration of head and pronotum metallic green with blue-purple overtones near margins. Elytra light brown. Mouthparts, mesoscutellum, legs, abdomen and ventral surface of body dark brown-black. Antennae dark brown except antennomeres 4–7 appearing yellowish brown due to the presence of yellow microtrichiae. Head slightly transverse, width: length ratio = 1.23. Dorsal surface of head with uniform dense polygon-shaped microsculpture, small punctures interspersed and medium to large size punctures throughout except medially. Ventral surface of head with transverse microsculpture, micropunctures and few large punctures along borders of gula and directly posterior to mandibles. Antennomeres 1–3 longer than wide; antennomere 4 shorter but wider than 3; antennomere 5 narrower than 6; antennomeres 6–7 subequal in size; antennomere 8 slightly wider than 7; antennomeres 8–10 subequal in size; antennomeres 5–11 serrate. Pronotum width: length ratio = 1.08, widest medially; with uniform dense polygon-shaped microsculpture; small punctures interspersed and medium to large size punctures throughout except medial line; medium to large size punctures also present around margin of pronotum but not in rows as is typical in other Xanthopygina. Mesoscutellum with polygon-shaped microsculpture and uniform small almost confluent punctures. Elytra longer than pronotum; with dense polygon-shaped microsculpture and uniform punctation consisted of medium-sized almost confluent punctures; sutures of elytra with 2–3 rows of micropunctures on each side. Abdominal tergites with dense transverse microsculpture and uniform small-sized punctures; punctures almost confluent except punctation less dense medially on tergites III–IV. Sternum with uniform dense punctuation consisted of small punctures; additional irregular row of larger punctures near posterior margin on sternites V–VII; sternum with transverse microsculpture. Male secondary sexual structures: posterior border of sternite VIII having deep V-shaped emargination medially; sternite IX with shallow U-shaped emargination. Aedeagus as in Figs 8–9; paramere separated anteriorly into two lobes; lobes slightly asymmetrical; paramere much shorter and narrower than median lobe; paramere without peg setae; in dorsal view each paramere lobe converging to rounded apex; in lateral view paramere curved upwards. Median lobe in dorsal view wide, converging to rounded apex; with single large dorsal tooth; in lateral view median lobe curved upwards to prominent tooth, then becoming much narrower and slightly curved downwards to rounded apex.

##### Etymology.

The species is named in honor of Mr David Sedaris, a prolific writer, as an appreciation for his fascination with the natural world. I spent many hours listening to Mr Sedaris’ audiobooks while preparing the specimens and the figures for this and other manuscripts.

##### Distribution.

Known from Bahía Blanca, Buenos Aires and Río Cuarto, Córdoba in Argentina.

##### Habitat.

Unknown; the climate in the areas mentioned above is humid subtropical to humid temperate. However, agricultural fields have replaced the original habitat in these localities.

##### Remarks.

It is rather remarkable that only two specimens are known for such a large species. I have examined the rove beetle collections of most major museums in North America and Europe but unfortunately I was not able to locate any additional specimens. One explanation might be that this species lives in refuse piles of ants or other Hymenoptera (see below for further discussion).

## Discussion

The Darwin specimen described in this paper as the holotype of *Darwinilus sedarisi* was given the specimen number 708 in the Insect Notes held by Darwin and Syms Covington (Darwin’s servant). Until now, this specimen was considered lost (or “not found”) according to Smith (1987) in the BMNH collection. Alternatively, Smith hypothesized that specimen 708 (or perhaps 3445, see Table 1) could have been present in the Field Museum (FMNH), Chicago, given that Kritsky (1981) mentioned a Darwin rove beetle specimen was present there. However, the presence of such specimen in FMNH is unlikely given that several Coleoptera curators (H. Dybas, H. Nelson, A. Newton, M. Thayer, R. Wenzel; Newton personal communication) were not aware of any such specimens. It is likely that several of the Darwin specimens considered “not found” in Table 1 have been curated to other parts of the collection in BMNH, presumably to where they taxonomically belong. However, that was not the case for specimen 708, which was found among unsorted Staphylinidae materials by my colleague A. Solodovnikov (personal communication). He transferred the specimen to the unidentified materials of the genus *Trigonopselaphus* as the best tentative placement, an act that allowed me to discover this specimen later on when I borrowed the *Trigonopselaphus* specimens from BMNH.

*Darwinilus* is superficially similar to *Trigonopselaphus* (due to the large habitus) but it is probably more closely related to the newly erected genus *Terataki* (Chatzimanolis 2013) and/or the genera *Haematodes* and *Weiserianum*. *Darwinilus* shares with *Terataki* and *Haematodes* similarities in the morphology of the head (hexagonal shape, position of ridges and sutures ventrally, and mouthpart morphology) and the partially divided parameres of the aedeagus. Given the fragile state of both specimens used to describe *Darwinilus*, more specimens are required to add this taxon to a molecular/morphological phylogeny of the subtribe (Chatzimanolis in preparation).

No data were available regarding the natural history of *Darwinilus sedarisi*. The genus *Weiserianum*, hypothesized to be related to *Darwinilus*, is known to be a myrmecophile (leafcutter ants; Scheerpeltz 1936). A few other large South American xanthopygines are known to occur with social Hymenoptera other than ants such as the species *Triacrus dilatus* Nordmann (in debris piles of *Stenopolybia vicina* (de Saussure), a vespid wasp; Wasmann 1902), but clearly natural history observations are needed to understand the biology of *Darwinilus sedarisi*. Future collecting expeditions should focus on gathering natural history information for *Darwinilus sedarisi* as well as better defining its distribution range. Presently, *Darwinilus sedarisi* is known from two localities (Bahía Blanca and Río Cuarto) in Argentina separated by several hundred kilometers. Although the exact date for the collecting event in Río Cuarto by Breuer is not known, it took place before 1935 since the Breuer collection was already in ZMHB by that time (Jaeger personal communication; Horn and Kahle 1935: 30). Much of the area between Bahía Blanca and Río Cuarto has been converted into agricultural fields and it is questionable if that is a suitable habitat for the species. One of course hopes that a newly described species is not already extinct. Perhaps more specimens of *Darwinilus* remain unsorted in Natural History Museums in North America, Europe or South America, and the publication of this paper will bring these specimens to light.

## Supplementary Material

XML Treatment for Darwinilus

XML Treatment for Darwinilus sedarisi
